# Blood lead levels and associated factors among preschool children in Ningbo, China: a cross-sectional study

**DOI:** 10.3389/fpubh.2026.1953335

**Published:** 2026-10-29

**Authors:** Fei Xu, Yefang Ke, Fei Li, Wenbo Lu, Wenyuan Liu

**Affiliations:** 1 Laboratory of Medicine, The Affiliated Women and Children's Hospital of University, Ningbo, China; 2 Department of Pediatrics, The Affiliated Women and Children's Hospital of University, Ningbo, China; 3 Ningbo Key Laboratory for the Prevention and Treatment of Embryo Original Diseases, The Affiliated Women and Children's Hospital of Ningbo University, Ningbo, China; 4 Ningbo Key Laboratory of Genomic Medicine and Birth Defects Prevention, The Affiliated Women and Children's Hospital of Ningbo University, Ningbo, China; 5 Department of Public Health, Zhejiang University School of Medicine, Hangzhou, China

**Keywords:** 25-hydroxyvitamin D, childhood lead poisoning, environmental exposure, health equity (MeSH), socioeconomic status

## Abstract

**Objective:**

This cross-sectional study aimed to investigate blood lead levels (BLLs) and their associated factors among preschool children (aged 3–7 years) in Ningbo, China. The findings may inform hypotheses for future prevention strategies against childhood lead exposure.

**Methods:**

Stratified Cluster sampling method was used to select three kindergartens in Ningbo, China, as study sites between January and May 2026. Questionnaire investigation about family, life, and behavioral conditions, venous blood samples collection and BLL detection are conducted.

**Results:**

In this study, an elevated BLL was defined as ≥35 μg/L. Of 595 children, 44 (7.39%) had elevated BLLs (≥35 μg/L; mean 19.43 μg/L). Before adjustment, lower 25-hydroxyvitamin D, town residence, factory proximity, coal gas use, and higher beverage frequency were associated with elevated BLLs (*p* < 0.001, *p* = 0.019, *p* = 0.022, *p* = 0.040, *p* < 0.001, respectively). After the adjustment, multivariable analysis showed each 1 μg/L increase in 25-hydroxyvitamin D (25(OH)D) was associated with 5% lower odds of elevated BLL (adjusted OR = 0.95, 95% CI: 0.90–0.99). Subgroup analyses revealed there is a significant interaction in males (OR = 0.92, 95% CI: 0.88–0.97) and town residents (OR = 0.92, 95% CI: 0.87–0.97).

**Conclusion:**

The level of 25(OH)D was significantly negatively correlated with the blood lead concentration (OR = 0.95, 95% CI: 0.90–0.99, *p* = 0.012). The level of 25(OH)D in the group with elevated blood lead was 19.5% lower than that in the normal group. The frequency of consuming sugary beverages was positively correlated with elevated blood lead levels (2–3 times per month: OR = 2.91; 2–3 times per week: OR = 4.72, *p* = 0.002). Living within 1,000 m of a factory, using liquefied gas, and living in a town were also associated with elevated blood lead levels. Subgroup analysis showed that this negative correlation was more significant in males (*p* = 0.001) and city children (*p* = 0.005). These cross-sectional associations suggest that 25(OH)D status and sugary beverage intake are correlated with BLLs in preschool children; however, causality cannot be inferred, and longitudinal or intervention studies are needed to clarify the direction of these relationships.

## 1 Introduction

Lead (Pb) is a naturally occurring element and also a common contaminant in human environments that can pose a serious threat to human health (1–3). Epidemiological studies have demonstrated that chronic lead exposure, even at low concentrations, adversely affects the hematopoietic, reproductive, renal, and central nervous systems, and causes irreversible neurodevelopmental impairment in children, particularly in younger age groups (4, 5). In 2012, the Centers for Disease Control and Prevention (CDC) reduced the blood lead reference value (BLRV) from 100 to 50 μg/L, and subsequently to 35 μg/L in 2021 (6, 7).

Lead poisoning has been recognized for centuries due to its extensive application and environmental persistence (8); however, lead exposure remains a critical global public health problem, especially in developing countries (9). As a developing country, China has experienced dramatic industrial and economic growth over the past decades and has become one of the largest lead producers and consumers worldwide.

Across different countries, reported sources of lead exposure in children vary based on national economic, social, and cultural conditions. Currently, children still face lead exposure risks from coal burning, petroleum fuel consumption, e-waste recycling piles (10, 11), ore and metal processing, lead-containing folk remedies (12, 13), and poor living environments (14–16).

China phased out leaded petrol in 2000 and has implemented various conventional public health strategies to reduce children’s lead exposure levels. However, there is still a lack of comprehensive understanding of lead exposure risks caused by regional differences, living environment, family circumstances, educational levels, feeding methods, and other sociodemographic factors. Additionally, since the international reference value for blood lead levels has been lowered to 35 μg/L, there remains a marked lack of research on blood lead levels within this range in China.

This cross-sectional study investigated the prevalence of elevated blood lead levels (BLLs, defined as ≥35 μg/L) among preschool children in the Ningbo area (Zhejiang Province, China) and analyzed its associated influencing factors. Although Ningbo City is a city, many of its suburban areas still retain rural characteristics. Additionally, the city has a China Petrochemical Economic and Technological Development Zone. With the petrochemical industry as the main industry, it covers refining and ethylene production, synthetic materials industry, polymer products industry, and fine chemical industry. These environmental factors may affect the risk of children being exposed to lead.

## 2 Methods

### 2.1 Study design and participants

A cross-sectional survey was conducted to examine blood lead levels (BLLs) and their influencing factors in children aged 3–7 years old in Ningbo, a city of Zhejiang Province located in southeast China. The study was approved by the institutional review boards of Ningbo Women and Children’s Hospital. All participants, as well as their parents or legal guardians, provided signed informed consent.

This study employed stratified cluster sampling method to select three kindergartens in Ningbo City. The sampling frame comprised all registered kindergartens in Haishu, Yinzhou, and Zhenhai districts, stratified by urbanicity (city center, suburban, rural). One kindergarten was randomly selected from each stratum using a random number generator. All eligible children present at the sampled kindergartens on the examination days were invited to participate. The sampling strategy was stratified cluster sampling: kindergartens served as the primary sampling units and were selected to represent diverse urbanicity levels (city center, suburban, rural) rather than through strict random selection of all eligible kindergartens. Only one kindergarten was selected per stratum, and all eligible children present on the examination days were invited. This approach improves logistical feasibility and demographic coverage, but the small number of clusters (*k* = 3) and the lack of random selection at the kindergarten level limit the generalizability of our findings to the entire preschool population of Ningbo, and may introduce selection bias if participating kindergartens differ systematically from non-participating ones.

Sample size was determined based on an expected elevated BLL prevalence of 10% (based on preliminary local data), with 80% power to detect an odds ratio of 0.90 per 1 μg/L increase in 25(OH)D (assuming SD = 12 μg/L, alpha = 0.05, two-tailed). The minimum required sample size was 442. We aimed to enroll 600 children to account for 15% non-response and provide adequate precision for multivariable modeling.

Inclusion criteria included all children present at the sampled kindergartens on the day of the physical examination who had no known chronic diseases or conditions that could affect blood lead levels. After obtaining informed consent from the children’s parents or guardians, a total of 595 children were enrolled in the study.

Exclusion criteria: known chronic kidney disease (may affect lead excretion), hemoglobinopathies (may affect hemoglobin-lead interactions), or current treatment for lead poisoning. Children with anemia were not excluded, as anemia may be a consequence of lead exposure.

Design effect: With a mean cluster size of approximately 198 children (595/3) and an intra-cluster correlation coefficient (ICC) for elevated BLL conservatively assumed to be 0.02, the design effect was estimated as 1 + (m − 1) × ICC = 1 + (198–1) × 0.02 ≈ 4.94. The effective sample size was therefore approximately 595/4.94 ≈ 120 children, which should be considered when interpreting the precision of our estimates. GEE models with kindergarten as the clustering variable and cluster-robust standard errors were used to account for this clustering.

### 2.2 Laboratory analysis and data collection

After written informed consent and assent were obtained from parents, a venous blood sample was collected from each participant. Before blood collection, participating children were instructed to fast for at least 8 h. Plain drinking water was permitted, but no food was allowed until blood collection was completed the next morning. As this study was conducted based on routine liver function tests for children and included an additional blood lead test, fasting blood collection was performed.

Peripheral venous blood samples were collected and stored in trace element tubes containing sodium heparin. To minimize discomfort and potential side effects, all sampling was performed by a skilled and experienced laboratory technician using single-use small-gauge needles. No cases of severe adverse effects requiring medical intervention were reported.

All blood samples were refrigerated immediately after collection, stored on-site in a 2–8 °C temperature-controlled container, and transported to the centralized Laboratory® for analysis. Lead concentrations in whole blood were measured using Inductively Coupled Plasma Mass Spectrometry (ICPMS) on the MS S1010 Trace Element Analyzer (Jiangxi Meikang Shengde Biotechnology Co., Ltd., Jiangxi, China). To ensure the daily precision of lead concentration measurements, quality control was consistently conducted using certified control material from Meikang (Jiangxi, China).

25(OH)D was measured using chemiluminescent immunoassay (CLIA) on the ADVIA Centaur XP Immunoassay System (Siemens Healthineers, Germany). The assay detects both 25(OH)D2 and 25(OH)D3 with cross-reactivity <3%. Analytical performance: LOD 4.0 μg/L; LOQ 8.0 μg/L; intra-assay CV 4.2–6.8%; inter-assay CV 6.5–9.1%; recovery 95–105%. Daily QC used two levels of certified control material (Siemens). Samples with 25(OH)D > 80 μg/L were diluted 1:2 and re-analyzed.

Lead concentrations in whole blood were measured by ICP-MS (MS S1010, Meikang Shengde, China). Analytical validation: LOD 0.1 μg/L; LOQ 0.3 μg/L; intra-assay CV 2.1–3.5% (*n* = 20 at 5 μg/L); inter-assay CV 3.8–5.2% (*n* = 20 across 5 batches); recovery 98.2–102.5% for spiked samples. Certified reference material (GBW09131, 10.8 ± 0.5 μg/L; GBW09132, 50.2 ± 2.1 μg/L) was analyzed with each batch; measured values were within certified ranges. Calibration used 6-point standard curve (0.5–100 μg/L, R^2^ > 0.999). Blank correction was applied using method blanks (*n* = 2 per batch). QC samples (*n* = 2 per 20 samples) were accepted if within ±10% of target; otherwise, the batch was repeated.

Finally, the researchers administered a structured interview questionnaire to identify possible influencing factors of exposure and possible sources of environmental lead. The questionnaire was developed by synthesizing items from the CDC Childhood Lead Poisoning Risk Assessment Questionnaire (17, 18), with several items adapted to the China context and tailored to the feasibility of the study. This questionnaire has undergone pre-tests, reliability evaluations, and other validations. Data collection was carried out using a pretested, which parents completed with the assistance of the research team. The questionnaire consisted of five main sections: (1) Demographics (e.g., age, gender, residential address (urban/rural)); (2) Parental socioeconomic information (e.g., education level, receipt of social welfare); (3) Environmental exposures (e.g., presence of a nearby factory); and (4) Behavioral and health history (e.g., parental smoking exposure). Missing responses (<3% for any item) were handled by multiple imputation (*n* = 20 imputations). The questionnaire form (in both Chinese and English) can be found in the Supplementary material.

All blood samples were collected between January and May 2026 (winter to spring). In Ningbo (latitude approximately 29.9°N), cutaneous vitamin D synthesis is minimal from November to March because of low solar elevation and short daytime duration; consequently, 25(OH)D concentrations in this study are expected to be lower than summer values, and the observed mean 25(OH)D (34.4 μg/L) should be interpreted as a cold-season estimate.

### 2.3 Statistical analysis

To account for potential correlation among children within the same kindergarten, we performed sensitivity analyses using generalized estimating equations (GEE) with an exchangeable working correlation matrix and kindergarten as the clustering variable. Cluster-robust standard errors were also calculated for the primary multivariable logistic regression model.

The dependent variable is children’s BLLs, which were categorized into normal and elevated BLLs following the guideline from the CDC, which has recommended a reference value of 35 μg/L since 2021 (5). In this study, blood lead levels (BLLs) were treated as the outcome and classified into two groups: <35 μg/L and ≥35 μg/L. Based on previous studies (19, 20) and directed acyclic graphs (Supplementary Figures S1, S2), we selected covariates associated with BLLs in our study. Covariates included age (continuous), gender (male or female), parents’ age (continuous), smoking by a household member (yes or no), 25-hydroxyvitamin D (continuous), hemoglobin (continuous), residential area type (urban or town), whether there are chemical factories within 1,000 meters of the residence (yes or no), floor (1–6, 7–12, 13–18, or >18 floors), the distance between the residence and the major motorized road (<20, 21–50, 51–100, 101–200, 210–500, or >500 m), the type of fuel used for cooking (nature gas, liquefied petroleum gas, or others), the frequency of beverage consumption (never, 2–3/months, or 2–3/week), the frequency of taking cod liver oil (never, 2–3/months, or 2–3/week), outdoor activity (<30, 30–60, >60 min) and sleep time (<8, 8–10, >10 h).

Age, hemoglobin and 25-hydroxyvitamin D were modeled as continuous variables, and all other variables were modeled as categorical variables. Data are presented as the mean ± standard deviation (SD) or median (interquartile range) for continuous variables, and as frequency (percentage) for categorical variables. We compared the baseline characteristics of the normal and elevated BLLs groups using t-tests, Mann–Whitney tests, or *χ*^2^ tests as appropriate. Univariate and multivariable logistic regression analyses were used to identify the association between 25(0H)D, area, factory, drink, fuel and BLLs. 25-hydroxyvitamin D was analyzed as a continuous variable. Living in an urban or town area, having a factory <1,000 m, the type of fuel and the frequency of drinking beverages were analyzed as categorical variables. Using the enter method, Model 1 was adjusted for age, gender and hemoglobin. Model 2 was adjusted for age, gender, hemoglobin, floor, road, take cod liver oil and outdoor activity.

To investigate the impact of various factors on the above-mentioned associations, interaction and stratified analyses were performed based on Model 2, stratified by gender, area, factory <1,000 m, and drinking beverages status, in accordance with the results of the univariate analysis (*p* < 0.05). All statistical analyses were performed using the software packages R 3.3.2^1^ and Free Statistics software version 2.1.1 A two-tailed test was adopted, and *p* < 0.05 was considered statistically significant.

## 3 Results

### 3.1 Baseline characteristics

A total of 595 children aged 3 to 7 years participated in this study, including 304 males (51.5%) and 291 females (48.9%). Table 1 outlines the demographic and clinical attributes of the participants involved in the study. Based on the reference value, 92.61% (*n* = 551) were identified as having normal BLLs, and 7.39% (*n* = 44) had elevated BLLs. The mean BLL was 19.43 μg/L (range: 9.25–69.2 μg/L).

**Table 1:** Baseline characteristics of the study population according to blood lead level category.

Characteristics	Total (*n* = 595)	Normal (*n* = 551)	Elevated (*n* = 44)	*p*
Hemoglobin, g/dL, Mean ± SD	12.43 ± 0.74	12.41 ± 0.74	12.61 ± 0.73	0.086
25(OH)D, μg/L, Mean ± SD	34.4 ± 11.6	34.9 ± 11.7	28.1 ± 9.4	<0.001
Age, year, Mean ± SD	5.4 ± 0.8	5.4 ± 0.8	5.5 ± 0.6	0.494
Gender, *n* (%)				0.084
Male	304 (51.1)	276 (50.1)	28 (63.6)	
Female	291 (48.9)	275 (49.9)	16 (36.4)	
Mother age, Mean ± SD	32.9 ± 4.4	33.0 ± 4.3	32.1 ± 5.6	0.201
Father age, Mean ± SD	34.8 ± 4.9	34.9 ± 4.8	34.2 ± 6.1	0.343
Common smoker, *n* (%)				0.838
Yes	190 (33.2)	177 (33.3)	13 (31.7)	
No	383 (66.8)	355 (66.7)	28 (68.3)	
Residential area, *n* (%)				0.019
City	219 (36.8)	210 (38.1)	9 (20.5)	
Town	376 (63.2)	341 (61.9)	35 (79.5)	
Factory < 1,000 m, *n* (%)				0.022
Yes	81 (13.6)	70 (12.7)	11 (25)	
No	514 (86.4)	481 (87.3)	33 (75)	
Floor, *n* (%)				0.053
1–6 floors	444 (75.5)	404 (74.3)	40 (90.9)	
7–12 floors	83 (14.1)	79 (14.5)	4 (9.1)	
13–18 floors	51 (8.7)	51 (9.4)	0 (0)	
>18 floors	10 (1.7)	10 (1.8)	0 (0)	
Road, *n* (%)				0.537
<20 m	92 (15.5)	83 (15.1)	9 (20.5)	
21–50 m	101 (17.0)	95 (17.2)	6 (13.6)	
51–100 m	118 (19.8)	113 (20.5)	5 (11.4)	
101–200 m	100 (16.8)	93 (16.9)	7 (15.9)	
201–500 m	98 (16.5)	90 (16.3)	8 (18.2)	
>500 m	86 (14.5)	77 (14)	9 (20.5)	
Fuel, *n* (%)				0.010
Nature gas	265 (44.5)	254 (46.0)	11 (25.0)	
Liquefied petroleum gas (LPG)	305 (51.3)	273 (49.5)	32 (72.7)	
Others	25 (3.9)	24 (4.4)	1 (2.3)	
Drink beverages, *n* (%)				< 0.001
Never	256 (43.0)	248 (45.0)	8 (18.2)	
2–3/month	259 (43.5)	235 (42.6)	24 (54.5)	
2–3/week	80 (13.4)	68 (12.3)	12 (27.3)	
Take cod liver oil, *n* (%)				0.077
Never	199 (34.3)	182 (33.8)	17 (39.5)	
2–3/month	144 (24.8)	128 (23.8)	16 (37.2)	
2–3/week	126 (21.7)	121 (22.5)	5 (11.6)	
Outdoor activity, *n* (%)				0.606
<30 min	74 (12.7)	67 (12.4)	7 (16.3)	
30–60 min	269 (46.1)	252 (46.6)	17 (39.5)	
>60 min	241 (41.3)	222 (41.0)	19 (44.2)	
Sleep time, *n* (%)				0.564
<8 h	32 (5.4)	29 (5.3)	3 (6.8)	
8–10 h	435 (73.9)	405 (74.3)	30 (68.2)	
>10 h	122 (20.7)	111 (20.4)	11 (25)	

Common smoke: ≥1 cigarette/day by any household member; factory is used as an alternative indicator to measure the potential level of environmental lead exposure, rather than to confirm the actual exposure situation; drink beverages: sugar-sweetened beverages consumed by the child; portion size not assessed; recall period: past month; major road: municipal road with ≥10,000 vehicles/day; cod liver oil: frequency of any vitamin D-containing supplement intake.

A comparison between the group with normal blood lead concentration and the group with elevated blood lead concentration revealed: there was a statistically significant difference in 25(OH)D levels between the group with normal blood lead levels and the group with elevated blood lead levels (*p* < 0.01). Moreover, the average level of 25(0H) D in children with elevated blood lead levels was significantly lower (28.1 ± 9.4 μg/L vs. 34.9 ± 11.7 μg/L, *p* < 0.001), which was 19.5% lower than that of children with normal blood lead levels. More participants were from town areas (63.2%) than from city areas (36.8%). Among children with elevated BLLs, 79.5% resided in town areas compared to 20.5% of those in city (*p* = 0.019). Thirteen point 6% (13.6%) of the children lived in houses <1,000 m of factories (mainly the petrochemical plant). Children with elevated BLLs were more likely to live within 1,000 m of factories (25.0% vs. 12.7%, *p* = 0.022). The types of fuel used in the kitchen were distributed as follows: 44.5% used natural gas, 51.3% used liquefied petroleum gas, and the remaining 4.2% used other types. Children with elevated BLLs had higher rates of liquefied petroleum gas use (72.7% vs. 49.5%) and lower rates of natural gas use (25.0% vs. 46.0%) compared to those with normal BLLs (*p* = 0.010). For beverage consumption frequency: 18.2% of children with elevated BLLs never drank beverages, compared to 45.0% of those with normal BLLs, while 54.5% of the elevated group drank 2–3 times per month (*p* < 0.001).

Several influencing factors were not found to be associated with elevated BLLs in our study population. There was no association with hemoglobin, age, gender, parents’ ages, family members smoking, the distance between the house and the main road with car service, living floor, outdoor activity time, sleep time, or the frequency of taking cod liver oil, calcium, iron, and zinc supplements.

### 3.2 Risk factors of children’s living environment associated with BLLs

Table 2 presents the results of the multivariate logistic regression models. Regardless of whether the relevant variables were adjusted or not, 25(OH)D was negatively correlated with blood lead concentration (*p* < 0.05). Living in a town was associated with higher unadjusted odds of elevated BLL (OR = 2.39, 95% CI: 1.02–4.85, Model 1), but this association was attenuated and non-significant after full adjustment for socioeconomic and environmental covariates (OR = 1.33, 95% CI: 0.54–3.27, *p* = 0.534, Model 2). The effect of factory proximity was attenuated to a marginal significance in Model 2 (*p* = 0.068). The intake of beverages is positively correlated with the lead content. The effect is robust: all three models are significant, and Model 2 still maintains a strong correlation after adjustment.

**Table 2:** Multivariable logistic regression analysis of the association between 25-hydroxyvitamin D, area, factory, drink and BLLs.

Variable	Total, *n*	Univariate OR	*p* value	Model1 OR	*p* value	Model2 OR	*p* value
25(OH)D, μg/L	595	0.94 (0.91–0.97)	<0.001	0.94 (0.91–0.97)	0.001	0.95 (0.91–0.99)	0.012
Residential area			0.023		0.044		0.534
City	219	1 (Ref)		1 (Ref)		1 (Ref)	
Town	376	2.39 (1.13–5.08)		2.23 (1.02–4.85)		1.33 (0.54–3.27)	
Factory <1,000 m			0.025		0.023		0.068
Yes	81	1 (Ref)		1 (Ref)		1 (Ref)	
No	514	0.44 (0.21–0.90)		0.43 (0.20–0.89)		0.47 (0.21–1.06)	
Drink beverages			<0.001		<0.001		0.001
Never	256	1 (Ref)		1 (Ref)		1 (Ref)	
2–3/month	259	3.17 (1.39–7.19)	0.006	3.16 (1.38–7.22)	0.006	2.91 (1.25–6.76)	0.013
2–3/week	80	5.47 (2.15–13.92)	<0.001	5.20 (2.03–13.34)	0.001	4.72 (1.77–12.6)	0.002

Association between 25-hydroxyvitamin D, residential area, factory <1,000 m, drink beverages and BLLs. Model 1: Age, gender, hemoglobin were adjusted, Model 2: Age, gender, hemoglobin, road, take cod liver oil, outdoor activity, floor were adjusted.

### 3.3 Subgroup analyses on the association between 25-hydroxyvitamin D levels and BLLs

We conducted subgroup analyses to examine the consistency of the association between 25(OH)D and blood lead levels across different populations (Figure 1). Overall, both before and after adjusting for confounding factors, 25(OH)D level was negatively correlated with blood lead level (unadjusted OR = 0.94, 95% CI: 0.91–0.97, *p* < 0.001; adjusted OR = 0.95, 95% CI: 0.90–0.99, *p* = 0.012). When stratified by gender, the association between 25(OH)D and blood lead reached statistical significance in males (OR = 0.92, 95% CI: 0.88–0.97, *p* = 0.001), but not in females (OR = 0.96, 95% CI: 0.92–1.01, *p* = 0.107). When stratified by residential area, the effect of 25(OH)D was statistically significant in children living in urban areas (OR = 0.92, 95% CI: 0.87–0.97, *p* = 0.005), but not in children living in rural areas (OR = 0.96, 95% CI: 0.89–1.03, *p* = 0.224). Among children living within a 1,000-m radius of a factory, the effect size estimate for 25(OH)D was imprecise (OR = 0.91, 95% CI: 0.81–1.02, *p* = 0.117), with its confidence interval crossing 1.0. When stratified by beverage intake frequency, the subgroup of children who never consumed beverages had an extremely small number of observations (*n* = 8, accounting for 3.1%), leading to severely insufficient statistical power for analysis of this subgroup. For the subgroups consuming beverages 2–3 times per month and 2–3 times per week, the effect of 25(OH)D was statistically significant (OR = 0.94, 95% CI: 0.90–0.99, *p* = 0.009) and marginally significant (OR = 0.91, 95% CI: 0.82–0.99, *p* = 0.049), respectively. However, the effect size estimates across all three groups were comparable; the observed differences in statistical significance were more likely caused by variations in statistical power rather than a true dose–response relationship.

**Figure 1:**
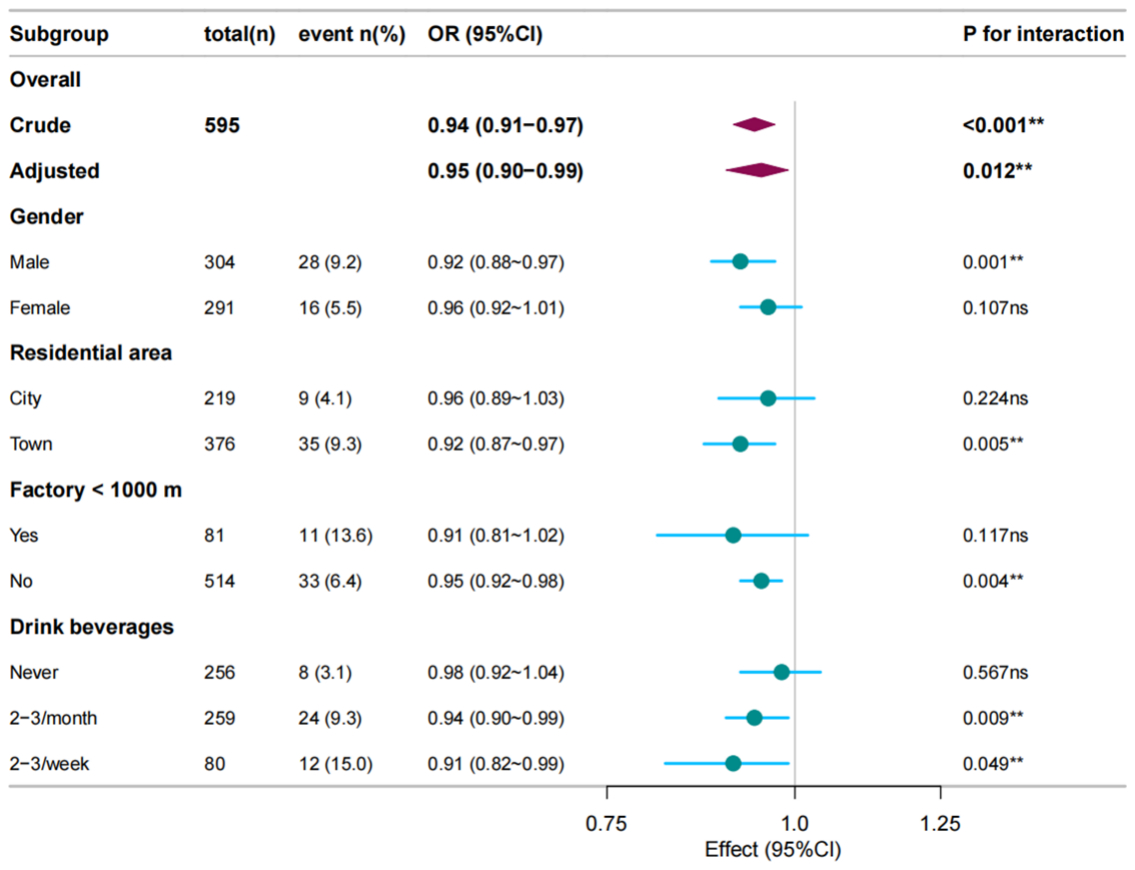
Effect size of 25-hydroxyvitamin D levels on blood lead in each subgroup. OR, odds ratio; CI, confidence interval.

## 4 Discussion

Lead exposure remains a major public health problem in China, as its extensive use in the manufacturing industry has grown alongside the country’s rapid economic development (12). This study assesses the prevalence and influence factors of elevated blood lead levels (BLLs) among preschool children (aged 3–7 years) in Ningbo, China. This population is particularly susceptible to lead exposure due to their frequent hand-to-mouth activity and heightened sensitivity to lead toxicity, stemming from their immature nervous systems.

This cross-sectional study was conducted in Ningbo, China, to measure BLLs among children aged 3–7 years and explore associated environmental and socioeconomic influence factors. We found that the mean BLL of the study population was 19.43 μg/L, with 7.39% of children having elevated BLLs. A 2021 national cross-sectional study covering 15 provinces and involving 32,543 children aged 0–6 years reported an overall weighted prevalence of 4.1%, but that study defined “blood lead elevation” as blood lead levels ≥50 μg/L (21). Although that study lacked comparative analysis against blood lead reference ranges, the mean BLL of children aged 36–84 months was 27.9 μg/L, which was higher than that of the same age group in our study. The proportion of elevated BLLs in our study (7.39%) was considerably lower than the 24.6% reported in industrial cities of Indonesia in 2022 (22). These levels were significantly lower than the average of 48.9 μg/L measured in other industrial pollution areas of Taizhou City, Zhejiang Province, where 43.9% of recorded BLLs exceeded 50 μg/L (23), and were comparable to the average value of 19.65 μg/L in developed cities such as Shanghai (14).

Our study examined sociodemographic and environmental factors that could be associated with lead exposure. The study has found no significant difference between mean lead levels in boys and girls, which confirms the findings from previous studies (14, 24). Though other studies have found that male students were 4–5 times more likely to have elevated BLLs than female students (25). Furthermore, our research indicates that children’s blood lead levels are associated with multiple environmental and behavioral factors: residential area (city vs. town), presence of chemical plants within a 1,000-meter radius, type of fuel used in household kitchens, and frequency of beverage consumption. Multiple studies conducted in China, Indonesia, India and Europe have identified highly similar patterns of risk factors across different exposure levels (26–30).

Multivariable logistic regression analysis showed that the associations of residential area (town vs. city) and factory proximity (<1,000 m) with elevated blood lead levels were statistically significant in univariate analysis and Model 1, but after adjusting for socioeconomic and environmental covariates in Model 2, the effects attenuated to non-significance (residential area: OR = 1.33, 95% CI: 0.54–3.27, *p* = 0.534; factory proximity: OR = 0.47, 95% CI: 0.21–1.06, *p* = 0.068). This finding suggests that the influence of these environmental factors on blood lead levels may be largely mediated by intermediate variables such as fuel type, dietary behaviors, and outdoor activity time, rather than representing independent direct effects.

The effect of fuel type (liquefied petroleum gas vs. natural gas) also attenuated in Model 2 (OR = 1.88, 95% CI: 0.80–4.42, *p* = 0.145), suggesting that this association may be partially mediated by variables including floor level, road distance, and outdoor activity time. Nevertheless, previous studies continue to support an association between fuel type and children’s blood lead levels. In a cross-sectional study from Indonesia, the use of non-clean fuels (including liquefied gas) in households was significantly associated with elevated blood lead levels in children (25). A national survey of children aged 0–6 years in China also reported that children from households using coal or liquefied gas as the primary cooking fuel had a significantly increased risk of elevated blood lead levels (23). Although in the single-factor analysis, the prevalence of elevated blood lead levels among children living within a distance of <1,000 m from the factory was 25.0%, while that of the control group was 12.7% (*p* = 0.022), after adjustment, the factor of factory distance no longer had statistical significance (*p* = 0.068), Mayans (31) pointed out in his review of the issue of lead poisoning in American children that even though the proportion of lead exposure sources in the environment on average is relatively small, in specific high-exposure subgroups, they may still pose a significant public health burden. Therefore, the public health significance of these environmental factors at the population level cannot be ignored, and further in-depth analysis can be conducted in subsequent studies.

Although the present study did not find a statistically significant association between gender and elevated blood lead levels (*p* = 0.084), we observed that the proportion of male children with elevated blood lead levels was higher than that of female children (63.6% vs. 36.4%), and in subgroup analyses, the protective effect of 25(OH)D against elevated blood lead levels reached statistical significance only in males (OR = 0.92, 95% CI: 0.88–0.97). This trend is consistent with previous findings that males have higher blood lead levels than females (25). Research has found that male junior high school students in Indonesia had a 5.62-fold higher risk of elevated blood lead levels than females (OR = 5.62, 95% CI: 2.00–15.81); the national survey of Chinese children aged 0–6 years similarly cited in its review the conclusion that the risk in males is 4–5 times that in females.

Potential explanations for the gender difference include: (1) behavioral differences—male children typically spend more time outdoors and engage in more vigorous activities, increasing opportunities for inhalation of dust and soil ingestion; (2) physiological differences—male children have higher bone turnover rates than females, leading to more rapid release of stored lead from bone (32); and (3) iron reserve differences—male children generally have lower iron stores than females, and iron deficiency increases intestinal lead absorption (33).

Another important finding of this study is the clear relationship between sugar-sweetened beverage consumption frequency and elevated blood lead levels: compared with never consumers, children who consumed beverages 2–3 times per month had a 2.91-fold increased risk of elevated blood lead levels (OR = 2.91, 95% CI: 1.25–6.76), while those who consumed beverages 2–3 times per week had a further increased risk of 4.72-fold (OR = 4.72, 95% CI: 1.77–12.6). This association remained robust across Model 1 and Model 2, and remained significant after adjusting for age, gender, hemoglobin, floor level, road distance, cod liver oil intake, and outdoor activity time.

This finding is supported by multiple studies. A national survey of Korean adolescents aged 12–19 years found that adolescents who frequently consumed sugar-sweetened beverages had significantly higher blood lead levels than non-consumers, and this association persisted after adjustment for age, gender, BMI, household income, and residential age (34). In the study of Mexican teenagers, the consumption of sugary beverages was found to be associated with higher levels of blood lead (35). Possible explanations for this association include: (1) nutritional substitution—frequent consumption of sugar-sweetened beverages may displace milk and other nutrient-dense foods, reducing dietary intake of calcium, iron, and zinc, which compete with lead for shared intestinal transporters such as DMT1 and thereby limit intestinal lead absorption (36); (2) fasting-state absorption—sugar-sweetened beverages are typically consumed between meals on an empty stomach, when gastrointestinal transit is accelerated and lead absorption is more efficient than in the postprandial state (37); and (3) residual container-related exposure—although lead-soldered cans have largely been phased out, imported or informally produced beverages may still occasionally contain lead from other sources (e.g., contaminated water, flavorings, or packaging materials), and this possibility cannot be fully excluded. Overall, the nutritional substitution hypothesis is the most plausible and best supported by the existing literature.

The most robust finding of this study is the inverse association between 25(OH)D and elevated blood lead levels, which remained consistent across univariate analysis (OR = 0.94, 95% CI: 0.91–0.97), Model 1 (OR = 0.94, 95% CI: 0.91–0.97), and Model 2 (OR = 0.95, 95% CI: 0.91–0.99). This finding is consistent with multiple epidemiological studies. Among the middle school students in Kuwait, the blood lead level was significantly negatively correlated with free 25(OH)D (38). In addition, a cross sectional study of lead exposed children in the southwestern region of China, there was a significant negative correlation between 25(OH)D and blood lead levels (39).

Study Limitations and Future Directions. This study has several limitations. First, the cross-sectional design precludes establishment of causality between 25(OH)D and BLLs, and reverse causation—where lead exposure causes 25(OH)D reduction—cannot be excluded. Second, dietary intake of calcium, iron, zinc, and vitamin D was not quantitatively assessed using a food-frequency questionnaire, and serum ferritin, transferrin saturation, and other iron-status biomarkers were not measured. Calcium, iron, and zinc compete with lead for the same intestinal transporters potentially omitting important confounding factors. Third, blood samples were collected only between January and May (winter to spring), a period of minimal cutaneous vitamin D synthesis at this latitude, and seasonal variations have a significant impact on 25(OH)D synthesis and the level of environmental lead exposure (40). This is a systematic limitation of the single-season sampling design, and multi-season studies are needed. Fourth, with only 44 elevated-BLL events and 8 covariates in the primary model (EPV = 5.5), the multivariable model may be at risk of overfitting; although LASSO sensitivity analyses produced consistent estimates, the confidence intervals should be interpreted with caution and the findings require confirmation in larger samples. Fifth, future studies could consider using local anesthesia (such as lidocaine cream) to further reduce the discomfort and anxiety of children, thereby making the methodology of the research more rigorous.

Future research should employ longitudinal designs or intervention trials to clarify whether vitamin D supplementation can effectively reduce BLLs; incorporate measurements of DBP, free 25(OH)D, and inflammatory indicators to elucidate the interactive mechanisms between vitamin D status and lead metabolism; and conduct multi-center studies to validate our findings. Future studies should consider systematic environmental exposure assessment tools such as the Green Page approach (41). From a public health perspective, assessing 25(OH)D nutritional status alongside routine blood lead screening for children living near industrial pollution sources, and considering 25(OH)D optimization as part of lead exposure prevention strategies, may have important practical implications.

## 5 Conclusion

This study included 595 children aged 3–7 years old. It was found that 7.39% had elevated blood lead levels. The level of 25(OH)D was significantly negatively correlated with the blood lead concentration (OR = 0.95, 95% CI: 0.91–0.99, *p* = 0.012). The level of 25(OH)D in the group with elevated blood lead was 19.5% lower than that in the normal group. The frequency of consuming sugary beverages may affect the increase in blood lead levels. (2–3 times per month: OR = 2.91; 2–3 times per week: OR = 4.72, *p* = 0.002). Living within 1,000 m of a factory, using liquefied gas, and living in a town were also may affect the blood lead levels. Subgroup analysis showed that this negative correlation was more significant in males (*p* = 0.001) and city children (*p* = 0.005). This indicates that 25(OH)D may be a factor influencing the low level of lead in children’s bodies; reducing the intake of sugary beverages and improving the living environment can both affect the level of lead in children’s bodies.

## Data Availability

The original contributions presented in the study are included in the article/Supplementary material, further inquiries can be directed to the corresponding author/s.

## Footnotes

1 http://www.Rproject.org, The R Foundation.
