# Supplementary material for: Blood lead levels and associated factors among preschool children in Ningbo, China: a cross-sectional study
Source: Front Public Health. 2026 Oct 29;14:1953335. doi: 10.3389/fpubh.2026.1953335 (PMC13755212; doi:10.3389/fpubh.2026.1953335)
Supplement: Supplementary file 1 [file Data_Sheet_1.docx]

Figure S1. Primary model: Adjusts for age, gender and dwelling environment.

Figure S2. Sensitivity model: Adjusts for age, gender, life style and dwelling environment.

Directed acyclic graph (DAG) depicting hypothesized causal relationships between 25-hydroxyvitamin D and elevated blood lead levels (BLLs ≥35 μg/L) in preschool children.

NODE COLORS (variable roles):

• Green with play icon (▶): Exposure variable — 25-hydroxyvitamin D

• Blue: Outcome variable — Elevated blood lead levels (BLLs ≥35 μg/L)

• Red: Confounders adjusted in the primary model — Age, Gender,

Residential area (city vs. town)

• Pink: Variable tested in sensitivity analyses only — Factory proximity

within 1000 m (collinear with residential area, VIF=3.9; replaces

residential area in Model B, not simultaneously adjusted)

• White: Variables explicitly NOT adjusted in any model

- Outdoor activity: Mediator (adjustment would underestimate total effect)

- Hemoglobin: Collider (adjustment would open a bias path:

iron status → hemoglobin ← BLLs)

• Gray: Unmeasured variables or variables not in primary consideration

- SES (socioeconomic status): Unmeasured; effects captured through

residential area proxy

- Take cod liver oil: Mediator; not adjusted in primary model

EDGES:

• Purple arrows: Hypothesized causal pathways based on temporal ordering

and biological plausibility

• Green bidirectional arrow: Reverse causation (elevated BLLs reduce

25-hydroxyvitamin D via inhibition of renal 1α-hydroxylase); this path

cannot be blocked in cross-sectional design

ASSUMPTION: All blood samples were collected within a 2-week period,

eliminating seasonal variation as a confounder.
