# Supplementary material for: Blood lead levels and associated factors among preschool children in Ningbo, China: a cross-sectional study
Source: Front Public Health. 2026 Oct 29;14:1953335. doi: 10.3389/fpubh.2026.1953335 (PMC13755212; doi:10.3389/fpubh.2026.1953335)
Supplement: Supplementary file 2 [file Data_Sheet_2.docx]

Fig S2 LASSO Cross-Validation Plot

Fig S3 LASSO coefficient profile polt

Fig S4 Nomogram constructed using four predictors selected by LASSO regression.
