# Supplementary material for: Blood lead levels and associated factors among preschool children in Ningbo, China: a cross-sectional study
Source: Front Public Health. 2026 Oct 29;14:1953335. doi: 10.3389/fpubh.2026.1953335 (PMC13755212; doi:10.3389/fpubh.2026.1953335)
Supplement: Supplementary file 3 [file Supplementary_file_1.docx]

Survey of Living Habits of Children in Ningbo Area in the Past 3 Months

{num}_________ {name}: _____________

General Information

Date of Birth: {birth} <yyyy/mm/dd> {Sex}: {sex}# 1=Male 2=Female

{Height}: {heigh}###cm {Weight}: {weight}##.#kg

Family Status:# 1=Only child 2=2 children 3=3 children 4=4 children or more

Family Income: {income}# 1=Below 50,000 RMB 2=50,000-100,000 RMB 3=100,000-300,000 RMB 4=300,000-500,000 RMB 5=Above 500,000 RMB

Mother: Age: {mom age}## years

Education: {momxl}# 1=Junior high school or below 2=High school 3=College 4=Bachelor's degree 5=Master's degree or above

Occupation: {momzy}# 1=Civil servant 2=Teacher 3=Medical staff 4=Business person 5=Company employee 6=Migrant worker 7=Farmer 8=Other____________

Smoking: {momsmoke}# 1=Yes 2=No

Father: Age: {father age}## years

Education: {fxl}# 1=Junior high school or below 2=High school 3=College 4=Bachelor's degree 5=Master's degree or above

Occupation: {fzy}# 1=Civil servant 2=Teacher 3=Medical staff 4=Business person 5=Company employee 6=Migrant worker 7=Farmer 8=Other____________

Smoking: {fsmoke}# 1=Yes 2=No

Does any co-resident smoke: {common smoker}# 1=Yes 2=No

Does any co-resident engage in lead-related work (e.g., printing industry, battery manufacturing, lead smelting, painting, ceramic production, etc.): {common lead}# 1=Yes 2=No

Living Environment

Area type: {area}# 1=Urban 2=Town 3=Coastal

Are there any factories within 1000 meters of the residence (smelting plants, printing plants, paint production plants, ceramic manufacturing plants, battery production plants, etc.): {factory}# 1=Yes 2=No

Housing floor: {floor}# 1=1-6 floors 2=7-12 floors 3=13-18 floors 4=Above 18 floors

Distance from residence to road with vehicle traffic(The definition of the variable is also explained in the questionnaire): {road}# 1=Less than 20 meters 2=21-50 meters 3=51-100 meters 4=101-200 meters 5=200-500 meters 6=More than 500 meters

Kitchen fuel: {fuel}# 1=Natural gas 2=Coal gas 3=Firewood stove 4=Other:____________

Living Habits

Hand washing: {wash}# 1=Almost never 2=1-5 times/day 3=>5 times/day

Finger sucking: {suck fingers}# 1=Almost never 2=1-5 times/day 3=>5 times/day

Nail biting: {gnaw nails}# 1=Almost never 2=1-5 times/day 3=>5 times/day

Pen biting: {bite pen}# 1=Almost never 2=1-5 times/day 3=>5 times/day

Reading colorful picture books, playing with colored clay, etc.: {read cartoon books}# 1=Almost never 2=1-5 times/day 3=>5 times/day

Feeding and Dietary Habits

Frequency of vitamin D supplementation in late pregnancy: {VD}# 1=Almost never 2=2-3 times/month 3=2-3 times/week 4=Daily

Breastfeeding: {breastfeeding}# 1=Less than 6 months 2=6-12 months 3=More than 12 months

Current daily milk intake: {amount of breastmilk}# 1=Less than 100ml 2=100-300ml 3=More than 300ml

Frequency of eating fried and puffed foods: {fried food} # 1=Almost never 2=2-3 times/month 3=2-3 times/week 4=Daily

Frequency of eating preserved duck eggs (pidan): {pidan}# 1=Almost never 2=2-3 times/month 3=2-3 times/week 4=Daily

Frequency of drinking commercial beverages: {drink}# 1=Almost never 2=2-3 times/month 3=2-3 times/week 4=Daily

Taking traditional Chinese medicine (TCM): {TCM}# 1=Almost never 2=Occasionally (1-3 times/year) 3=Frequently (every quarter)

Frequency of supplementing cod liver oil, calcium, iron, and zinc: {cod liver oil}# 1=Almost never 2=2-3 times/month 3=2-3 times/week 4=Daily

Daily outdoor activity time: {outdoor activities}# 1=Less than 30 minutes 2=30-60 minutes 3=More than 60 minutes

Daily sleep duration: {sleep time}# 1=Less than 8 hours 2=8-10 hours 3=More than 10 hours

Frequency of growing pains (joint pain): {Growth arthralgia}# 1=Almost never 2=2-3 times/month 3=2-3 times/week 4=Daily

Clinical Manifestations {Clinical}#

1=None 2=Hyperactivity 3=Anemia 4=Other:____________

Blood lead: Blood lead (Reference range: <100 μg/L)

Hemoglobin: Hemoglobin, unit g/L, reference range as follows

[Image: Hemoglobin reference range table]

VD: 25-hydroxyvitamin D (Reference range: 30-100 ng/mL)
