# Supplementary material for: Blood lead levels and associated factors among preschool children in Ningbo, China: a cross-sectional study
Source: Front Public Health. 2026 Oct 29;14:1953335. doi: 10.3389/fpubh.2026.1953335 (PMC13755212; doi:10.3389/fpubh.2026.1953335)
Supplement: Supplementary file 4 [file Supplementary_file_2.docx]

宁波地区儿童近3个月生活习惯调查表

{num}_________	{name}：___________

一般情况

出生日期：{birth} <yyyy/mm/dd>	{性别}：{sex}# 1=男	2=女 {身高}：{heigh}###cm	{体重}：{weight}##.#kg

家庭状况:#	1=独生子女	2=2个孩子	3=3个孩子	4=4个孩子及以上

家庭收入：{income}#	1=5万以下	2=5-10万	3=10-30万	4=30-50万	5=50万以上

母亲：年龄：{mom age}##岁

学历：{momxl}# 1=初中及以下	2=高中	3=大专	4=本科	5=硕士及以上

职业：{momzy}# 1=公务员 2=教师 3=医务人员 4=商人 5=公司职员	6=务工人员	7=农民 8=其他______________

吸烟：{momsmoke}# 1=是 2=否

父亲：年龄：{father age}##岁

学历：{fxl}# 1=初中及以下	2=高中	3= 大专	4=本科	5=硕士及以上

职业：{fzy}# 1=公务员 2=教师 3=医务人员 4=商人 5=公司职员	6=务工人员	7=农民 8=其他______________

吸烟：{fsmoke}# 1=是 2=否

是否有共同居住者吸烟：{common somker}#	1=有	2=无

是否有共同居住者从事铅相关工作（比如：印刷业、电池制造业、铅冶炼业、油漆工、陶瓷制作等）：{common lead}#	1=有	2=无

居住环境

区域属性：{area}#	1=城市 2=乡镇 3=沿海

居住房屋周边（1000米内）是否有工厂（冶炼厂、印刷厂、油漆生产类厂、陶瓷制造厂、电池生产厂等）：{factory}#	1=有	2=无

住房楼层：{floor}#	1=1-6层	2=7-12层	3=13-18层	4=18层以上

住户离通汽车的马路距离（日通行量≥10,000辆的市政道路）：{road}# 1=小于20米 2=21-50米 3=51-100米 4=101-200米 5=200-500米 6=500米以上

厨房燃料：{fuel}# 1=天然气 2=煤气 3=灶火 4=其他：______________

生活习惯

洗手：{wash}#	1=几乎不	2=1-5次/天	3=>5次/天

吸手指：{suck fingers}#	1=几乎不	2=1-5次/天	3=>5次/天

啃指甲：{gnaw nails}#	1=几乎不	2=1-5次/天	3=>5次/天

咬笔头：{bite pen}#	1=几乎不	2=1-5次/天	3=>5次/天

看彩色绘本、彩色胶泥等：{read cartoon books}#	1=几乎不	2=1-5次/天	3=>5次/天

喂养和饮食习惯

母亲怀孕后期补充维生素D频率：{VD}# 1=几乎不 2=2-3次/月 3=2-3次/周	4=每天

母乳喂养：{breastfeeding}#	1=6个月以内	2=6-12个月	3=大于12个月

目前每日奶量：{amount of breastmilk}#	1=小于100ml	2=100-300ml 3=大于300ml

吃油炸和膨化类食品频率：{fried food} #	1=几乎不 2=2-3次/月	3=2-3次/周	4=每天

吃松花蛋食品频率：{pidan}# 1=几乎不	2=2-3次/月	3=2-3次/周	4=每天

喝商品饮料频率： {drink}# 1=几乎不	2=2-3次/月	3=2-3次/周	4=每天

喝中药：{TCM}#	1=几乎不 2=偶尔（一年1-3次） 3=经常（每季度都喝）

补充鱼肝油、钙、铁、锌的频率：{cod liver oil}# 1=几乎不 2=2-3次/月	3=2-3次/周	4=每天

每天户外活动时间：{outdoor activities}# 1=小于30分钟	2=30-60分钟	3=大于60分钟

每天睡眠时间状况：{sleep time}# 1=小于8小时	2=8-10小时	3=10小时

生长关节痛频率：{Growth arthralgia}# 1=几乎不 2=2-3次/月 3=2-3次/周 4=每天

临床表现{Clinical}#

1=无	2=多动	3=贫血	4=其他：_____________

Bloodlead:血铅（参考区间：<100ug/L）

Hemoglobin:血红蛋白，单位g/L，参考区间如下

VD: 25羟维生素D（参考区间30-100ng/ml）
